# Quantification of cure for pharmacodynamic models of antimalarial drugs: Deterministic versus stochastic approaches

**DOI:** 10.1002/bcp.70340

**Published:** 2025-11-08

**Authors:** Meg K. Tully, Robert J. Commons, Julie A. Simpson, David J. Price

**Affiliations:** ^1^ Centre for Epidemiology and Biostatistics, Melbourne School of Population and Global Health University of Melbourne Melbourne Victoria Australia; ^2^ Global and Tropical Health Division Menzies School of Health Research and Charles Darwin University Darwin Northern Territory Australia; ^3^ General and Subspecialty Medicine Grampians Health—Ballarat Ballarat VIC Australia; ^4^ Centre for Tropical Medicine and Global Health, Nuffield Department of Medicine University of Oxford Oxford UK; ^5^ Department of Infectious Diseases University of Melbourne at the Peter Doherty Institute for Infection and Immunity Melbourne Victoria Australia

**Keywords:** infectious diseases, modelling and simulation, pharmacokinetic–pharmacodynamic

## Abstract

In silico pharmacokinetic–pharmacodynamic (PK–PD) models are used to inform dose optimisation in antimalarial drug development. Here the PK–PD models capture the change in parasite count over time because of exposure to antimalarial drug(s). For current deterministic PD models, simulated parasite numbers decline proportionally during treatment asymptotically approaching zero, requiring a cure threshold to be selected. We implemented an alternative stochastic model, where parasite‐time profiles were simulated using a binomial function with an hourly parasite survival probability. This probabilistic model enables a time of exact cure to be calculated, when zero parasites remain. The 28‐day cure rates predicted by the deterministic and stochastic models for multiple antimalarial treatment strategies were equivalent, with an absolute difference ranging from −0.8% to 3.1% (mean of 0.48%, positive values indicate higher stochastic model cure rates). This confirms that the standard deterministic method is an adequate proxy for the underlying stochastic process of parasite death during antimalarial treatment.

**What is already known about this subject?**

- In silico pharmacokinetic–pharmacodynamic models of malaria infection typically represent parasite death from antimalarial treatment as a continuous percentage of parasites killed.
- This approach requires the selection of an arbitrary threshold on the parasite count at which a simulated patient is declared cured.

**What this study adds?**

- We tested an alternative model for simulating parasite‐time profiles, which uses probabilities to calculate the number of parasites, rather than the proportion, that are killed each hour following antimalarial treatment.
- Both methods produced essentially identical 28‐day cure rates.
- Therefore, the current percentage‐kill method is a suitable approximation for the more realistic, probabilistic model.

## 1 INTRODUCTION

There are approximately 235 million cases of malaria each year caused by *Plasmodium falciparum*,^1^ with most patients treated with an artemisinin‐based combination therapy (ACT).^2^ The emergence and spread of artemisinin resistant parasites has reduced the efficacy of these first‐line treatments, highlighting the urgent need for new antimalarial treatments.^3^

Drug development and registration is a protracted process requiring preclinical laboratory studies, and phase I, II and III clinical trials, prior to comprehensive regulatory review.^4^ In silico pharmacokinetic–pharmacodynamic (PK‐PD) modelling is an important tool to streamline the drug development pipeline. PD models estimate the change in the number of parasites over time within an individual patient because of antimalarial treatment. Key antimalarial treatment outcomes, such as 28‐day cure rates, can be calculated from simulated parasite‐time profiles, informing dose optimisation and the design of early phase antimalarial clinical trials.

Mechanistic PD models of *P. falciparum* infection capture the underlying biology of antimalarial drug effects and changes in parasite growth. During a malaria infection, parasites invade red blood cells, progressing from ring stages to trophozoites to schizonts 48 h later, before parasite replication and reinvasion of new red blood cells. Most antimalarial drugs target only a relatively narrow window of the parasite developmental stages (e.g. mefloquine targets parasites aged 18–40 h^5^), although artemisinins and the new antimalarials, cipargamin and ganaplacide, can kill parasites aged 6–44 h.^6^, ^7^ Incorporating fine‐scale parasite age dynamics into PK–PD modelling of antimalarial drugs improves the predictive power of these models.

Mechanistic *P. falciparum* PD models (developed and implemented in^8^, ^9^, ^10^, ^11^, ^12^, ^13^, ^14^), capture the antimalarial drug action on parasite age using an hourly, discrete‐time framework. The antimalarial treatment's age‐specific parasiticidal effect is represented in the model as a proportion of vulnerable parasites killed each hour. Iteratively removing a percentage of the parasite population simulates a parasite burden that asymptotically approaches zero. This deterministic method requires a cutoff threshold (e.g. one parasite or one parasite per millilitre) to be selected to define cure for a simulated patient.

Although this percentage parasite death rate is straightforward and easy to implement, the selection of a cure threshold is not clearly justified. Therefore, a more biologically plausible approach may be to consider the parasite population as strictly an integer value, where cure is achieved at a parasite count of exactly zero. To ensure a discrete population count, the drug effect can be employed as a per‐parasite hourly probability of death, via a binomial distribution, thus introducing a probabilistic function into the model. In comparison to the current deterministic method, this new stochastic method removes the need to select a cutoff value to define cure.

This report implemented and compared two PK–PD models of malaria infection, the current deterministic method and an alternative stochastic method. We performed a comprehensive simulation study, calculating 28‐day cure rates of a cohort of hypothetical patients and repeated the comparison for a selection of antimalarial drug resistance scenarios, to determine how model specification impacted simulation outcomes.

## 2 METHODS

We conducted a simulation study in R (version 4.4.0^15^) to compare the cure rates for an ACT between a stochastic PD model and a deterministic PD model for *P. falciparum* malaria.

One thousand PK profiles were simulated for a paediatric population treated with WHO weight‐based dosing regimens of the widely used ACTs artemether–lumefantrine and dihydroartemisinin–piperaquine.^16^ Patient weights were sampled from the Severe Malaria in African Children Network's study dataset of paediatric malaria patients.^17^ The PK models used for all four drugs were based on those previously published, with parameter distributions sourced from existing PK studies (see Tables S1.1 and S1.2).

**FIGURE 1:**
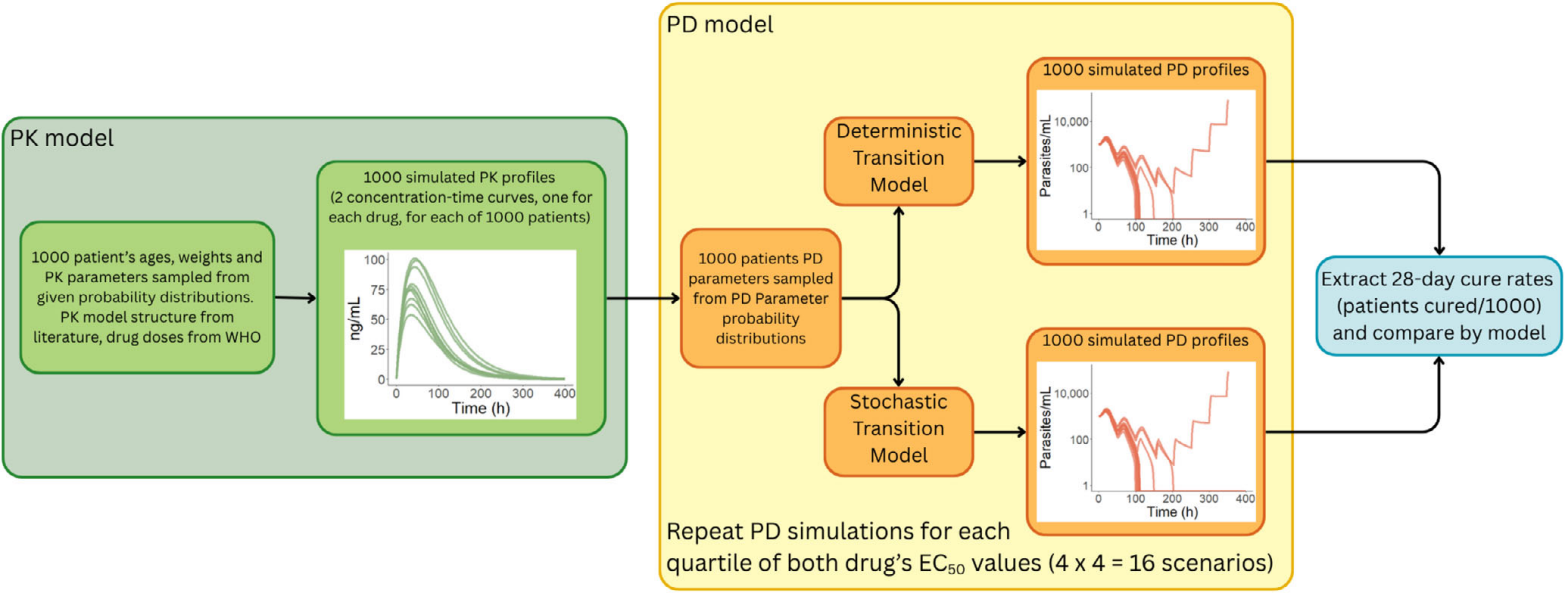
A simplified schematic of the pharmacokinetic–pharmacodynamic (PK–PD) simulation process used to compare two methods of determining parasite cure (deterministic and stochastic) following antimalarial treatment. Each antimalarial drug's pre‐defined $EC_{50}$ ranges (artemether: [4.38, 46.20], lumefantrine: [1.75, 2331.60], dihydroartemisinin: [1.44, 532.05], and piperaquine: [11.56, 94.19] all in ng/ml) were broken into equal quartiles, to represent no‐, low‐, medium‐ and high‐levels of antimalarial drug resistance. The simulation scenarios for each treatment span each combination of these $EC_{50}$ ranges, resulting in 16 total scenarios for artemether–lumefantrine and another 16 scenarios for dihydroartemisinin–piperaquine, all tested with both methods.

Parasite‐time profiles were calculated by combing a PD model of parasite growth and death with the simulated drug‐concentration profiles (Figure 1 and Table S1.3). This PD model uses discrete hourly transitions, such that the number of parasites in age group a at time t are given by:

(1) $$N\left(a,t\right)=\{\begin{array}{l} N\left(a-1,t-1\right)\times \left(1-0.5\left(E\left(a-1,t\right)+E\left(a-1,t-1\right)\right)\right),\;2\leq a\leq 48\\ N\left(T_{\max},t-1\right)\times \left(1-0.5\left(E\left(48,t\right)+E\left(48,t-1\right)\right)\right)\times \mathrm{PMF},\;a=1. \end{array}$$

Here, the number of parasites *a* hours old at time t, $N\;\left(a,t\right)$, is the number 1 hour *younger* $\left(a-1\right)$, 1 hour *before* $\left(t-1\right)$, multiplied by the age‐specific survival rate (1 − mean death rate over the previous hour^18^). The number of 1‐hour old parasites is given by the product of the number of 48 h‐old parasites, the age‐specific survival rate and the parasite multiplication factor ( PMF). The PMF represents the average number of infected red blood cells derived from one schizont at the end of its lifecycle (i.e. the average number of new merozoites released multiplied by the red blood cell invasion efficiency).

Drug d has a parasite kill effect, $E_{d}\left(a,t\right)$, that acts only on specific stages of the parasite—defined by $W_{d}$ (e.g. for artemisinin $W_{\mathrm{ART}}=\left\{6,7,\ldots ,44\right\}$ hours and for lumefantrine $W_{\mathrm{LF}}=\left\{18,19,\ldots ,40\right\}$). This death rate is a function of the drug concentration at time $t,\;C_{d}\left(t\right)$, that is, the PK model output, the maximum killing effect of the drug ( $E_{\max,d}$), the concentration for which 50% of that maximum killing effect is achieved ( ${\mathrm{EC}}_{50,d}$), and the sigmoidicity (sharpness) of the concentration–effect curve ( $\gamma_{d}$):

(2) $$E_{d}\left(a,t\right)=\left\{\begin{array}{c} E_{\max,d}\left(\frac{C_{d}{\left(t\right)}^{\gamma_{d}}}{C_{d}{\left(t\right)}^{\gamma_{d}}+{{\mathrm{EC}}_{50,d}}^{\gamma_{d}}}\right),\;a\in W_{d}\\ \;0,\;\text{else} \end{array}.\right.$$

For artemether–lumefantrine (and ACTs in general), the two drugs are assumed to have different modes of action, and therefore act independently such that $E\left(a,t\right)=E_{d1}\left(a,t\right)+E_{d2}\left(a,t\right)$ ‐ $E_{d1}\left(a,t\right)E_{d2}\left(a,t\right)$.

The typical deterministic approach^8^, ^9^, ^10^, ^11^, ^12^, ^13^, ^14^ is to implement $E\left(a,t\right)$ by calculating a percentage of parasites that survive each hourly transition (Equation 1). Thus, the population declines during treatment, asymptotically approaching, but never reaching, zero (i.e. ‘cure’). In practice, a threshold number of parasites is selected, and when the total number of parasites drops below this value, the simulated patient is classified as cured.

Under an alternative stochastic method, applying $E\left(a,t\right)$ as a per‐parasite hourly probability of death (instead of an hourly percentage killed), we classify a patient as cured when their parasite count declines to exactly zero parasites. We can adjust Equation (1) to incorporate the probability of death per parasite (which typically represents a single parasite in the body but could also indicate one parasite per millilitre or per microlitre; see S4) via a binomial distribution, where the number of Bernoulli processes is given by the number of parasites at each age, and the probability of survival is a function of the drug effect on parasites of that age, at that time. Therefore, under the stochastic model, the number of parasites a hours old at time t is given by:

(3) $$N\left(a,t\right)=\{\begin{array}{l} \text{Binom}\left(N\left(a-1,t-1\right),1-0.5\left(E\left(a-1,t\right)+E\left(a-1,t-1\right)\right)\right)\;2\leq a\leq T_{\max}\\ \text{Binom}\left(N\left(T_{\max},t-1\right)\times \mathrm{PMF},1-0.5\left(E\left(T_{\max},t\right)+E\left(T_{\max},t-1\right)\right)\right),\;a=1. \end{array}$$

The fixed set of 1000 simulated PK profiles for artemether–lumefantrine were used to simulate parasite profiles using the two models for 16 total scenarios, by varying the $EC_{50}$ for each drug to mimic four levels of varying drug‐resistance and testing all combinations (see Figure 1 caption or Table S1.4). This process was repeated for dihydroartemisinin–piperaquine, creating another 16 resistance scenarios. The primary outcome was 28‐day cure rate, defined as the percentage of the 1000 hypothetical paediatric patients classified as cured by day 28 of follow‐up. A threshold of one parasite was used for cure in the deterministic model. The secondary outcomes included (1) simulation efficiency (i.e. computation time) and (2) 28‐day cure rates for simulations where cure threshold values were shifted, by running the simulations on different scales (total parasite burden, number of parasites per millilitre or number of parasites per microlitre). Thus, if the cure threshold is set to one unit for all simulations, this scaling creates three adjusted cutoff thresholds of one, 1000 or 1000 000 total parasites, respectively, assuming approximately 1 l median paediatric blood volume; see S4. These theoretical thresholds are chosen purely for the purpose of model comparison and are not intended to be used in practice.

Monotherapy 28‐day cure rates for artemether and lumefantrine treatments were also compared across both models as a sensitivity analysis to further evaluate outcome robustness to different drug scenarios.

## 3 RESULTS

**FIGURE 2:**
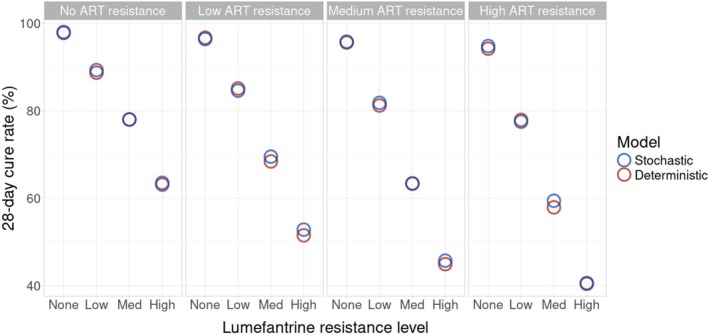
Simulated 28‐day cure rates using a deterministic and stochastic pharmacokinetic–pharmacodynamic (PK–PD) model, for a 1000‐patient paediatric cohort treated with WHO standard doses of artemether (ART)–lumefantrine, across four levels of resistance for each drug (see Table S1.4).

Two sets of 1000 drug‐concentration profiles were simulated, one for each combination therapy. These fixed pharmacokinetic profiles were then used to simulate parasitaemia profiles for each of the 16 resistance scenarios per treatment. The 28‐day cure rates calculated using the standard deterministic method compared with the stochastic method were essentially equal across all artemether–lumefantrine resistance scenarios (mean 0.17% higher cure rates for the stochastic model, range [−0.6%, 1.5%] Figure 2) and dihydroartemisinin–piperaquine scenarios (mean 0.76% higher cure rates for the stochastic model, range [−0.8%, 3.1%] Figure S6.1).

Resistance to artemether had a comparatively mild effect on simulated cure rates when compared to resistance to lumefantrine. With no resistance to either drug, the simulated cure rates were 98.0% for the stochastic and 97.8% for the deterministic method. Simulations with high lumefantrine mono‐resistance decreased cure rates to 63.5% and 63.1%, compared with simulations with high artemether mono‐resistance where the cure rates declined to 94.2% and 94.8%. Dihydroartemisinin–piperaquine results were also consistent (Figure S6.1), with piperaquine resistance influencing cure rates more significantly than dihydroartemisinin resistance.

Computational runtime differences between the methods were negligible for the same input scenarios: a mean of 1.66 min (16%) longer for the stochastic compared with the deterministic method (see S3). Of note, the stochastic model was implemented for parasite counts below one million for computational feasibility. This modification was validated by demonstrating it produced the same 28‐day cure rates across all scenarios, when applied below one, 10 or 100 million parasites (see Figure S3.1).

As expected, calculated cure‐rates were highly dependent on the scale of the simulated parasite counts. For artemether–lumefantrine, a simulation using the total number of parasites in the body as the scale (which is standard), with a threshold for cure of one parasite in the body, produced a mean 28‐day cure rate of 74.2% (averaging across both methods and all 16 resistance scenarios). An identical simulation using a parasite per millilitre of blood scale for the PD model parameter ipl, initial parasite load at treatment, with a threshold for cure of 1 parasite per millilitre (which is equal to 1000 parasites total, assuming 1 l as an approximate median blood volume for the SMAC cohort) produced a mean 28‐day cure rate of 89.6%. At a per microlitre scale (i.e. 1/μl cutoff = 1 000 000 total for 1 l blood volume), the mean 28‐3day cure rate was further inflated by almost 10% to 98.1% (see S4). Results for dihydroartemisinin–piperaquine remained consistent with those for artemether–lumefantrine.

The sensitivity analysis of hypothetical monotherapy of artemether and lumefantrine confirmed that the stochastic and deterministic models also produced highly consistent 28‐day cure rates across all drug resistance scenarios (see S5 and Figure S5.1).

## 4 DISCUSSION

We implemented a stochastic PK–PD model of antimalarial treatment for *P. falciparum* infection, which restricted parasite population counts to whole numbers via binomial transitions, and enabled the quantification of a specific time of cure. The 28‐day cure rates calculated from simulated data were equivalent between the novel stochastic model and the existing deterministic model. Results were consistent across a range of drug resistance scenarios for artemether–lumefantrine and dihydroartemisinin–piperaquine, validating the use of the deterministic method as an accurate approximation of the more biologically plausible stochastic method.

One limitation of the deterministic approach is that the selection of a suitable threshold for declaring cure is not clearly justified but can impact PD simulation outcomes. Significant differences in simulated cure rates were observed when the cure cutoff values were varied (i.e. one parasite/millilitre blood compared to one parasite total, see S4, Table S4.1 and Figure S4.1). Although the standard approach is to simulate total parasite numbers with a cure cutoff of 1, these results highlight the importance of clearly reporting the threshold chosen for a cured infection and specifying an appropriate threshold to avoid over estimating 28‐day cure rates. The deterministic and stochastic PK–PD models were compared in a simulation setting, with resistance represented via increases in $EC_{50}$ (one plausible resistance mechanism). These comparisons span a broad range of antimalarial drug resistance scenarios with day 28 cure rates varying between 0.5% and 100%. In line with previous in silico ACT simulations,^11^ partner drug resistance impacted cure rates more significantly than artemisinin resistance. This is also consistent with evidence from Cambodia, where artemisinin–resistant regions experienced cure rate reductions following the emergence of resistance to the partner drug.^19^ Furthermore, the hypothetical patient population was based on the body weights from the Severe Malaria in African Children Network's study dataset, ensuring realistic simulations for children infected with *P. falciparum* in malaria endemic regions.

Drug concentrations and parasite profiles were simulated for artemether–lumefantrine and dihydroartemisinin–piperaquine as well as for artemether‐only and lumefantrine‐only hypothetical treatments in a sensitivity analysis (see S5 and Figures S5.1, S6 and S6.1). Calculated 28‐day cure rates were consistent between the stochastic and deterministic models for all scenarios, suggesting the findings are generalisable to antimalarial drugs with similar pharmacokinetic and pharmacodynamic properties. We hypothesise that slow acting drugs applied over a long treatment period are likely to be more sensitive to the additional variability induced by the stochastic model. In the event that the simulated time‐to‐cure is close to the point of clinical outcome (i.e. day 28), an exploration of how the stochastic model's variability compares to the deterministic model may be warranted.

Sun and colleagues^20^ previously described a stochastic‐transition model of malaria infection, noting that fine‐scale stochastic phenomena, specifically spontaneous extinction of the parasite population (i.e. because of immune response) cannot be adequately captured by a purely deterministic model. Our results, show that for dose‐optimisation simulations, a deterministic model suitably approximates the probabilistic process of parasite death. This remains true even when the probability of cure is low, and fine‐scale dynamics are likely to have a greater effect on the simulated outcome.

In conclusion, this simulation study demonstrates that the standard deterministic PK–PD model for *P. falciparum* malaria, which uses a cure threshold of one total parasite in the body, provides robust results when compared to a more computationally intensive stochastic alternative. A conceptual limitation of the deterministic approach is the required selection of a suitable cure threshold. Here we demonstrate that as expected, this cutoff value (or equivalently, the simulation scale) will significantly impact model outputs. Therefore, we recommend transparent reporting of all simulation details, including the definition of cure that was used, in order to assure model reproducibility.

## AUTHOR CONTRIBUTIONS

Robert J Commons, Julie A Simpson and David J Price designed the simulation study and reviewed and edited the manuscript. Meg K Tully designed and performed the simulation study and wrote the manuscript.

## CONFLICT OF INTEREST STATEMENT

All authors declare that they have no competing interests.

## Supporting information


**Table S1.1:** **Definitions of pharmacokinetic (PK) model parameters.**
Table S1.2: Pharmacokinetic parameter distributions.
Table S1.3: Definitions of pharmacodynamic (PD) model parameters.
Table S1.4: Pharmacodynamic parameter distributions.
**Figure S3.1:** **Comparison of simulated 28‐day cure rates for PK‐PD model of artemether‐lumefantrine, using a hybrid method (deterministic model for large numbers of parasites, and stochastic model below a cutoff) with a threshold of 1 million, 10 million and 100 million. Simulations were run over 16 resistance scenarios for robustness. Here we see the threshold of 1 million was suitable, as the results do not materially differ from each other.**
Table S4.1: Scaling Scenario Inputs.
Figure S4.1: Simulated 28‐day cure rates for 1000 paediatric patients treated with artemether‐lumefantrine, by level of resistance to each drug, and the simulation cure threshold.
Figure S5.1 Simulated 28‐day cure rates for 1000 paediatric patients treated with hypothetical artemether monotherapy (Panel A) and lumefantrine monotherapy (Panel B), by level of resistance and the simulation cure threshold.
Figure S6.1: Simulated 28‐day cure rates for 1000 paediatric patients treated with dihydroartemisinin‐piperaquine, by level of resistance to each drug, and the simulation cure threshold of parasite profiles.

## Data Availability

Data sharing not applicable to this article as no datasets were generated or analysed during the current study.
