# Supplementary material for: Quantification of cure for pharmacodynamic models of antimalarial drugs: Deterministic versus stochastic approaches
Source: Br J Clin Pharmacol. 2025 Nov 8;92(1):317–22. doi: 10.1002/bcp.70340 (PMC12746395; doi:10.1002/bcp.70340)
Supplement: Supplementary file 1 — Table S1.1: Definitions of pharmacokinetic (PK) model parameters. Table S1.2: Pharmacokinetic parameter distributions. Table S1.3: Definitions of pharmacodynamic (PD) model parameters. Table S1.4: Pharmacodynamic parameter distributions. Figure S3.1: Comparison of simulated 28‐day cure rates for PK‐PD model of artemether‐lumefantrine, using a hybrid method (deterministic model for large numbers of parasites, and stochastic model below a cutoff) with a threshold of 1 million, 10 million and 100 million. Simulations were run over 16 resistance scenarios for robustness. Here we see the threshold of 1 million was suitable, as the results do not materially differ from each other. Table S4.1: Scaling Scenario Inputs. Figure S4.1: Simulated 28‐day cure rates for 1000 paediatric patients treated with artemether‐lumefantrine, by level of resistance to each drug, and the simulation cure threshold. Figure S5.1 Simulated 28‐day cure rates for 1000 paediatric patients treated with hypothetical artemether monotherapy (Panel A) and lumefantrine monotherapy (Panel B), by level of resistance and the simulation cure threshold. Figure S6.1: Simulated 28‐day cure rates for 1000 paediatric patients treated with dihydroartemisinin‐piperaquine, by level of resistance to each drug, and the simulation cure threshold of parasite profiles. [file BCP-92-317-s001.docx]

Supplemental Text: Quantification of cure for pharmacodynamic models of antimalarial drugs: deterministic versus stochastic approaches

**Author list & affiliations**

Meg K Tully^1^, Robert J Commons^2,3^, Julie A Simpson^1,4#^, David J Price^1,5#^

1. Centre for Epidemiology and Biostatistics, Melbourne School of Population and Global Health, University of Melbourne, Melbourne, VIC, Australia
2. Global and Tropical Health Division, Menzies School of Health Research and Charles Darwin University, Darwin, NT, Australia
3. General and Subspecialty Medicine, Grampians Health—Ballarat, Ballarat, VIC, Australia
4. Centre for Tropical Medicine and Global Health, Nuffield Department of Medicine, University of Oxford, United Kingdom
5. Department of Infectious Diseases, University of Melbourne, at the Peter Doherty Institute for Infection and Immunity, Melbourne, VIC, Australia

**S1: Pharmacokinetic-Pharmacodynamic (PK-PD) model parameters and definitions**

Artemether was simulated using a one-compartment model, such that:

$$\frac{dA_{g}}{dt}=-k_{a}A_{g}$$

$\frac{dA}{dt}=k_{a}A_{g}-\frac{Cl}{V}A$.

Lumefantrine was simulated using a two-compartment model, such that:

$$\frac{dA_{g}}{dt}=-k_{a}A_{g}$$

$$\frac{dA_{c}}{dt}=k_{a}A_{g}-\left( \frac{Cl}{V_{c}}+\frac{Q}{V_{c}} \right)A_{c}+\frac{Q}{V_{p}}A_{p}$$

$\frac{dA_{p}}{dt}=\frac{Q}{V_{c}}A_{c}-\frac{Q}{V_{p}}A_{p}$.

**Table S1.1: Definitions of pharmacokinetic (PK) model parameters**

| **Symbol** | **Definition** |
| --- | --- |
| $k_{a}$ | Absorption rate constant (/hours). |
| $Cl$ | Elimination clearance rate (litres/hour). |
| $Q$ | Inter-compartmental clearance rate (litres/hour). |
| $V_{c}$ | Volume in the main/central compartment for a two-compartment model (litres).  $V$ for a one-compartment model. |
| $V_{p}$ | Volume in the peripheral compartment for a two-compartment model (litres). |
| $A_{g}$ | Amount of drug in the gut (milligrams). At $t=0, A_{g}=dose$ (in mg). |
| $A_{c}$ | Amount of drug in the main/central compartment for a two-compartment model (milligrams). $A$ for a one-compartment model. |
| $A_{p}$ | Amount of drug in the peripheral compartment for a two-compartment model (milligrams). |

**Table S1.2: Pharmacokinetic parameter distributions**

Artemether-lumefantrine parameters sourced from [1] while. dihydroartemisinin and piperaquine parameters are from [2] and [3] respectively, all standardised to a 50kg patient (see allometric scaling, S2). All in the form; individual patient values, $\theta_{i}$ ~ $\theta*\exp\left( \omega\right),\omega\sim N(0, \sigma^{2})$. Where $\sigma$ represents between-subject-variance (BSV). Within-patient correlations of the parameters were assumed to be negligible for the purpose of this simulation study. Weight-based dosing schedules sourced from WHO guidelines [4].

| **Compound** | **Parameter** | **Individual mean parameter value (**$\boldsymbol{\theta)}$ | **BSV (**$\boldsymbol{\sigma}$**)** |
| --- | --- | --- | --- |
| Artemether | $k_{a}$ (/hr) | 0.37 | 0.63 |
|  | $Cl$ (L/hr) | 180 | 0.50 |
|  | $V$ (L) | 217 | 0.4 |
| Lumefantrine | $k_{a}$ (/hr) | 0.17 | 0.52 |
|  | $Cl$ (L/hr) | 7.04 | 0.16 |
|  | $V_{c}$ (L) | 103 | 0.4 |
|  | $Q$ (L/hr) | 4.08 | 0.4 |
|  | $V_{p}$ (L) | 272 | 0.4 |
| Dihydroartemisinin | $k_{a}$ (/hr) | 0.82 | 0.265 |
|  | $Cl$ (L/hr) | 50.5 | 0.224 |
|  | $V$ (L) | 41.4 | 0.5 |
| Piperaquine | $k_{a}$ (/hr) | 0.717 | 1.68 |
|  | $Cl$ (L/hr) | 69 | 0.42 |
|  | $V_{c}$ (L) | 9021 | 1.01 |
|  | $Q$ (L/hr) | 136.5 | 0.85 |
|  | $V_{p}$ (L) | 25000 | 0.50 |

**Table S1.3: Definitions of pharmacodynamic (PD) model parameters**

| **Symbol** | **Definition** |
| --- | --- |
| *ipl* | Initial parasite load; Parasites per µL at time 0 (generally time of treatment initiation). |
| $\mu_{ipl}$ | Initial mean parasite age (hours). |
| $\sigma_{ipl}$ | Standard deviation of the age distribution of the initial parasite load (hours). |
| $PMF$ | Parasite multiplication factor. Average number of parasites released by a ruptured schizont at the end of the life cycle that successfully invade red blood cells. |
| $E_{max}$ | Maximal hourly killing rate of the drug (percentage). |
| $EC_{50}$ | *In vivo* drug concentration when killing rate is 50% of $E_{max}$ (ng/mL). |
| $\gamma$ | Slope of *in vivo* drug concentration-effect curve. |
| $\boldsymbol{W}_{\boldsymbol{D}}$ | Parasite age range window for which the drug $D$ is active (hours). |

**Table S1.4: Pharmacodynamic parameter distributions.**

For an individual patient’s parameter value $\theta_{i}$;

*Normal:* $\theta_{i}\sim N\left( \mu,\sigma^{2} \right),$ *Lognormal:* $\theta_{i}=\theta\times e^{\omega_{i}}, \omega\sim N\left( 0,\sigma^{2} \right),$ *Uniform:* $\theta_{i}\sim U[a,b]$*, Constant:* $\theta_{i}=\mu$*, Triangular:* $\theta_{i} \sim\left\{ \begin{aligned} \frac{2(\theta_{i}-a)}{(c-a)(b-a)}, a\leq\theta_{i}\leq b \\ \frac{2(c-\theta_{i})}{(c-a)(c-b)}, b<\theta_{i}\leq c \\ 0, \theta_{i}<a, \theta_{i}>c \end{aligned} \right.$

| **Parameter** | **Drug** | **Distribution** | **Values** | **Source/Justification** |
| --- | --- | --- | --- | --- |
| $ipl$ (/µL) | - | Lognormal | $\theta=20,000 ,$  $\sigma=2$,  $a=1,000$,  $b=200,000$ | Limits for uncomplicated malaria parasitaemia levels [4]. Note: $ipl$ was sampled with a ‘bounded’ lognormal, where iteratively, any individual patient values sampled outside the given bounds [a,b] were resampled, until all values were inside the upper and lower limits. |
| $\mu_{ipl}$ (hrs) | - | Uniform | $a$ = 1, $b$ = 15 | Treatment is generally commenced when most parasites are circulating (causing symptoms). Parasites begin to sequester (stop circulating) at around 16 hours old [5] so the centre of the distribution should be slightly younger than 16. |
| $\sigma_{ipl}$ | - | Lognormal | $\theta=$4,  $\sigma=0.1$ | Symptomatic infections are assumed synchronous (minimal spread in parasite age distribution), so a small range was selected, which enabled most of the burden to be circulating at one time. |
| $PMF$ | - | Triangular | $b=10,$  $a=8, c=12$ | [6] |
| $T_{max}$ (hrs) | - | Constant | $\mu$ = 48 | [5] |
| $E_{max}$ | Artemether | Triangular | $b=0.25$  $a$ = 0.15,  $c$ = 0.35 | Parasite reduction ratios ($PRR$) values from [7] transformed into suitable $E_{max}$ values based on code from S. Dini, using methods in supplement of [8]. |
|  | Lumefantrine |  | $b=$ 0.33  $a$ = 0.16,  $c$ = 0.50 |  |
|  | Dihydroartemisinin |  | $b=$ 0.69  $a$ = 0.49,  $c$ = 0.59 | Calculated in [8] from results in [9] |
|  | Piperaquine |  | $b=$ 0.35  $a$ = 0.19,  $c$ = 0.50 | Calculated in [8] from results in [10] |
| $EC_{50}$ (ng/ml) | Artemether | Uniform, ranges divided into 4 even segments for testing resistance scenarios^†^ | $a=4.38,$  $b=46.20$ | Estimated in [7] ^††^ |
|  | Lumefantrine |  | $a=1.75,$  $b=2331.60$ |  |
|  | Dihydroartemisinin |  | $a=1.44$  $b= 532.05$ |  |
|  | Piperaquine |  | $a=11.56,$  $b= 94.19$ |  |
| $\gamma$ | Artemether | Lognormal | $\theta=4.61 ,$  $\sigma=0.31$ | [11] |
|  | Lumefantrine |  | $\theta= 2.24,$  $\sigma= 0.58$ |  |
|  | Dihydroartemisinin |  | $\theta= 1.31,$  $\sigma= 0.65$ |  |
|  | Piperaquine |  | $\theta= 1.35,$  $\sigma= 0.66$ |  |
| $\boldsymbol{W}_{\boldsymbol{ART}}$ | Artemether | - | 6-44 hours | [12] |
| $\boldsymbol{W}_{\boldsymbol{LF}}$ | Lumefantrine |  | 18-40 hours | [13] |
| $\boldsymbol{W}_{\boldsymbol{ART}}$ | Dihydroartemisinin |  | 6-44 hours | [12] |
| $\boldsymbol{W}_{\boldsymbol{LF}}$ | Piperaquine |  | 12-36 hours | [13] |

^†^Four levels of drug resistance – no, low, medium and high – were specified for each drug as the quartiles of their ranges. For artemether and lumefantrine this is [4.38, 46.20] and [1.75, 2331.60] ng/mL, giving (U[4.38, 14.84], U[14.84, 25.95], U[25.29, 35.75], U[35.75, 46.20]) and (U[1.75, 584.21], U[584.21, 1166.67], U[1166.67, 1749.14], U[1749.14, 2331.60]) distributions respectively. For dihydroartemisinin and piperaquine this is [1.44, 532.1] and [11.56, 94.19] ng/mL, giving (U[1.44, 134.09], U[134.09, 266.75] U[266.75, 399.40], U[388.40, 523.05], and (U[11.56, 32.22], U[32.22, 52.88], U[52.88, 73.53], U[73.53, 94.19]) distributions respectively.

^††^“The lower limit of the distribution of EC50 was chosen to be the in vitro IC50 (the concentration that inhibits the growth of parasites by 50%) of free drug, obtained by adjusting for the in vitro drug bindings. The higher limit was chosen to be half of the maximum drug concentration of the median of the PK profile.” [7]

**S2: Allometric Scaling**

To incorporate the effect of patient body weight on pharmacokinetic (PK) parameters, each simulated parameter was transformed to $\theta_{i}^{*}$, prior to use in the simulation model, as follows;

$\theta_{i}^{*}= \theta_{i}\times\left( \frac{x_{i}}{x_{pop}} \right)^{\kappa}$,

where;

- $\theta_{i}$is the patient’s PK parameter value, sampled from an appropriate distribution in Table S1.2
- $\theta_{i}^{*}$ is the updated parameter which is used in the model simulations
- $x_{i}$ is the patient’s weight in kg
- $x_{pop}$ is the median cohort weight in kilograms, (specifically, the cohort from which the PK parameter distributions in use were estimated, not the cohort used in the simulation)
- $\kappa$ is defined as follows:$\kappa= \left\{ \begin{aligned} 0.75, \theta_{i}\in\{Cl, Q\} \\ 1, \theta_{i}\in\{V_{c}, V_{p}\} \end{aligned} \right.$ .

**S3: Hybrid Method**

Due to the more complex computations required, the simulation runtime under the stochastic method was significantly higher than the standard deterministic method, and often infeasibly long. To improve efficiency we applied the stochastic method in a hybrid model; using the simpler deterministic approach when the number of parasites exceeded a threshold, and the stochastic method if it was below. Thresholds of 1, 10 and 100 million parasites were tested on artemether-lumefantrine and found equivalent, consequently the 1 million threshold was selected for shortest runtime.


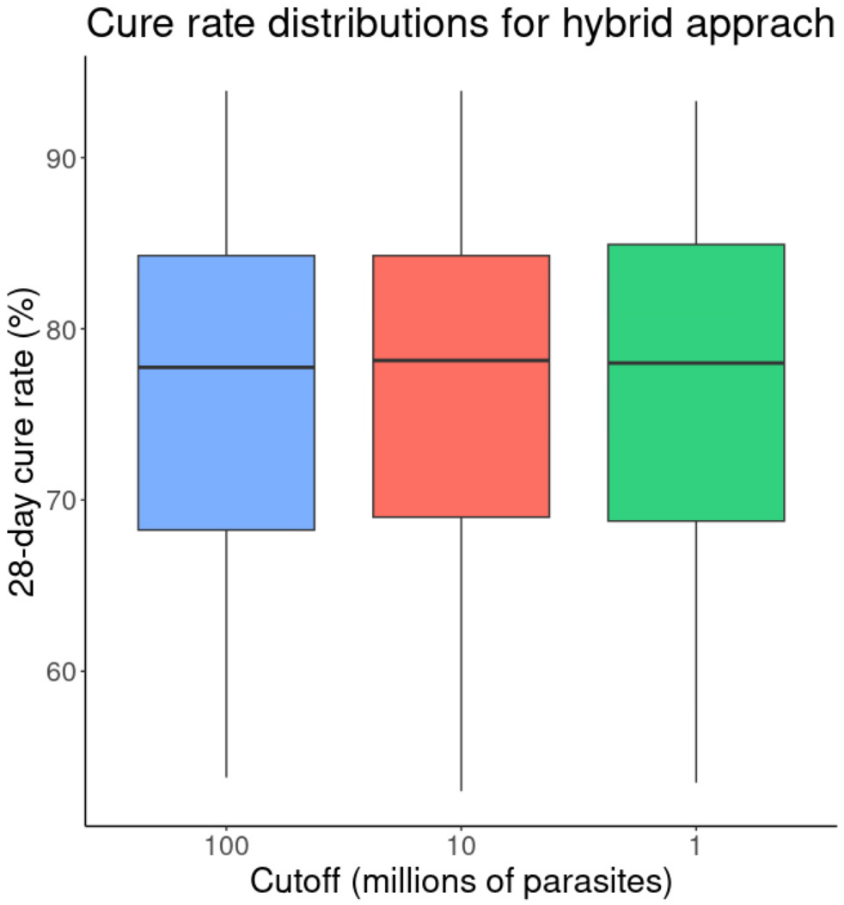


**Figure S3.1:** Comparison of simulated 28-day cure rates for PK-PD model of artemether-lumefantrine, using a hybrid method (deterministic model for large numbers of parasites, and stochastic model below a cutoff) with a threshold of 1 million, 10 million and 100 million. Simulations were run over 16 resistance scenarios for robustness. Here we see the threshold of 1 million was suitable, as the results do not materially differ from each other.

**S4: Parasite Population Scale Impact**

PD simulations are usually run on a total parasite burden scale, if a different scale is used (e.g., the number of parasites per millilitre of circulating blood volume), the threshold at which we determine ‘cure’ is also scaled. Measured parasite burdens are almost always reported in parasite per millilitre or microlitre scale, which is transformed into a total parasite burden in order to simulate total parasite counts. This therefore implies accurate calculation of total blood volume for an individual (e.g., often approximated by 5L for an adult) may be relevant to simulating an accurate probability of cure.

**Table S4.1: Scaling Scenario Inputs**

| **Scale Scenario** | **Implementation Method** | **Cure Threshold**  **(in total parasite numbers)** |
| --- | --- | --- |
| Total (standard) | Multiply initial parasite load (/$\mu$L) by 1,000,000, the approximate median total blood volume for this cohort in microlitres:  $1,000,000\times ipl/\mu L=ipl$ total | 1 total parasite |
| /mL | Multiply initial parasite load (/$\mu$L) by a factor of 1,000, to convert to millilitres:  $1,000\times ipl/\mu L=ipl/$mL | 1 Parasite/mL = 1,000 total parasites |
| /µL | Leave initial parasite load on original scale | 1 Parasite/µL = 1,000,000 total parasites |

Assuming approximately 1L total blood volume for a median child in the Severe Malaria in African Children Network’s study dataset ($12kg\times80ml/kg=960mL\approx1L,$ [14, 15])

**Figure S4.1: Simulated 28-day cure rates for 1000 paediatric patients treated with artemether-lumefantrine, by level of resistance to each drug, and the simulation cure threshold.**

**
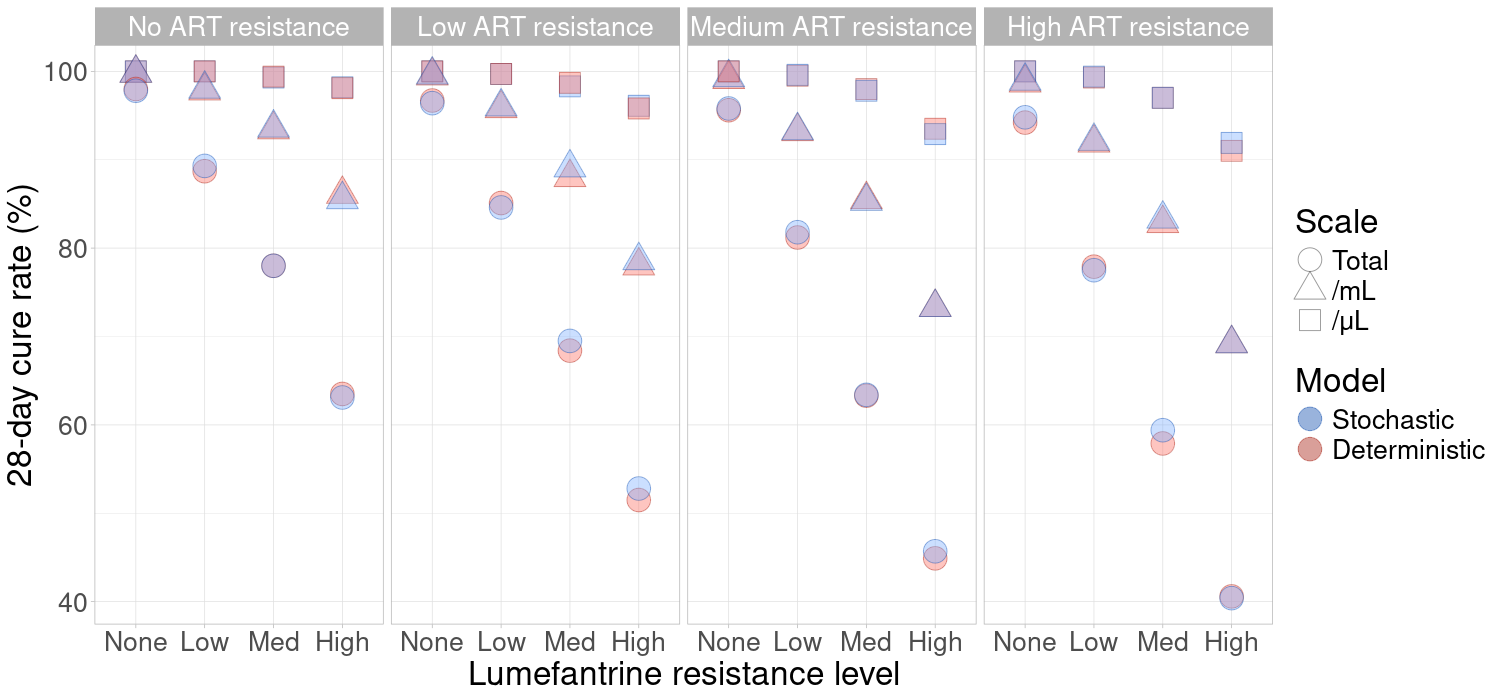
**

**S5: Monotherapy sensitivity analyses**

To further evaluate the robustness of our model comparison, we repeated the above simulations for a hypothetical scenario where only artemether and lumefantrine were administered as monotherapies. Both drugs were simulated using the same WHO dosing schedules as for artemether-lumefantrine combination therapy, across four resistance levels and three simulation scales identical to the main analysis (12 total scenarios per drug).

As in the primary results, both the stochastic and deterministic approaches produced consistent 28-day cure rate estimates for all scenarios. Mean absolute differences of 0.25% (range [-0.7%, 1.6%]), and 0.45% (range [-0.3%, 2.3%]) were calculated across the 12 artemether and 12 lumefantrine monotherapy scenarios respectively, where positive values indicate higher 28-day cure rates for the stochastic model.

**Figure S5.1 Simulated 28-day cure rates for 1000 paediatric patients treated with hypothetical artemether monotherapy (Panel A) and lumefantrine monotherapy (Panel B), by level of resistance and the simulation cure threshold.**


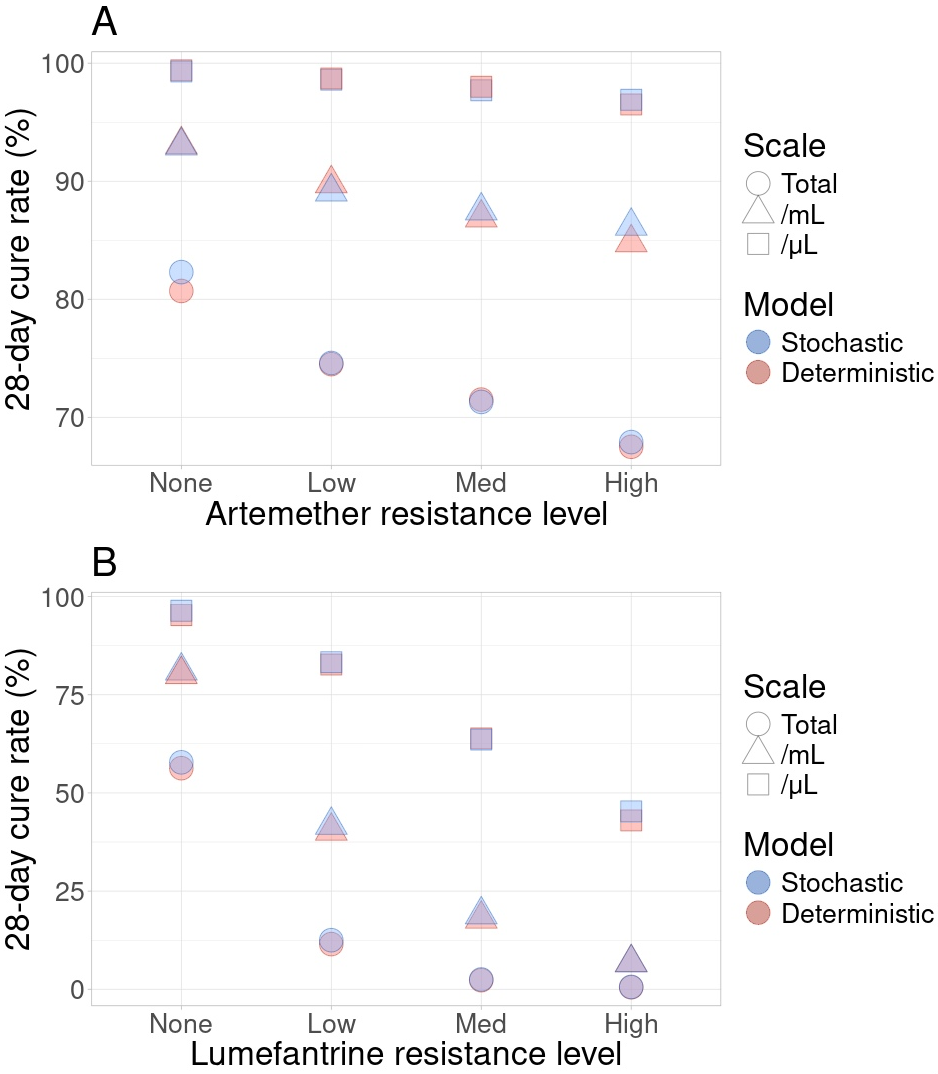


**S6: Dihydroartemisinin-piperaquine analysis**

To evaluate the generalisability of the results to other similar-actingfrequently prescribed antimalarial drugs, we also repeated the simulations for all 16 resistance scenarios and 3 scales (16 x 3 = 48 comparisons) when testingfor a second artemisinin-combination therapy; dihydroartemisinin (DHA)-piperaquine. Of note, piperaquine has a longer elimination time than lumefantrine and therefore represents significantly altered pharmacokinetic dynamics (. both are modelled here with two-compartment models, however the peripheral compartment volume is centred at 2500L for piperaquine compared to 272L for lumefantrine, demonstrating delayed clearance) compared to artemether-lumefantrine.

As in the primary results, both the stochastic and deterministic approaches produced consistent 28-day cure rate estimates for all scenarios. A mean absolute difference of 0.76% (range [-0.8%, 3.1%]), was calculated across the 48 scenarios, where positive values indicate higher 28-day cure rates for the stochastic model.

**Figure S6.1: Simulated 28-day cure rates for 1000 paediatric patients treated with dihydroartemisinin-piperaquine, by level of resistance to each drug, and the simulation cure threshold of parasite profiles.**


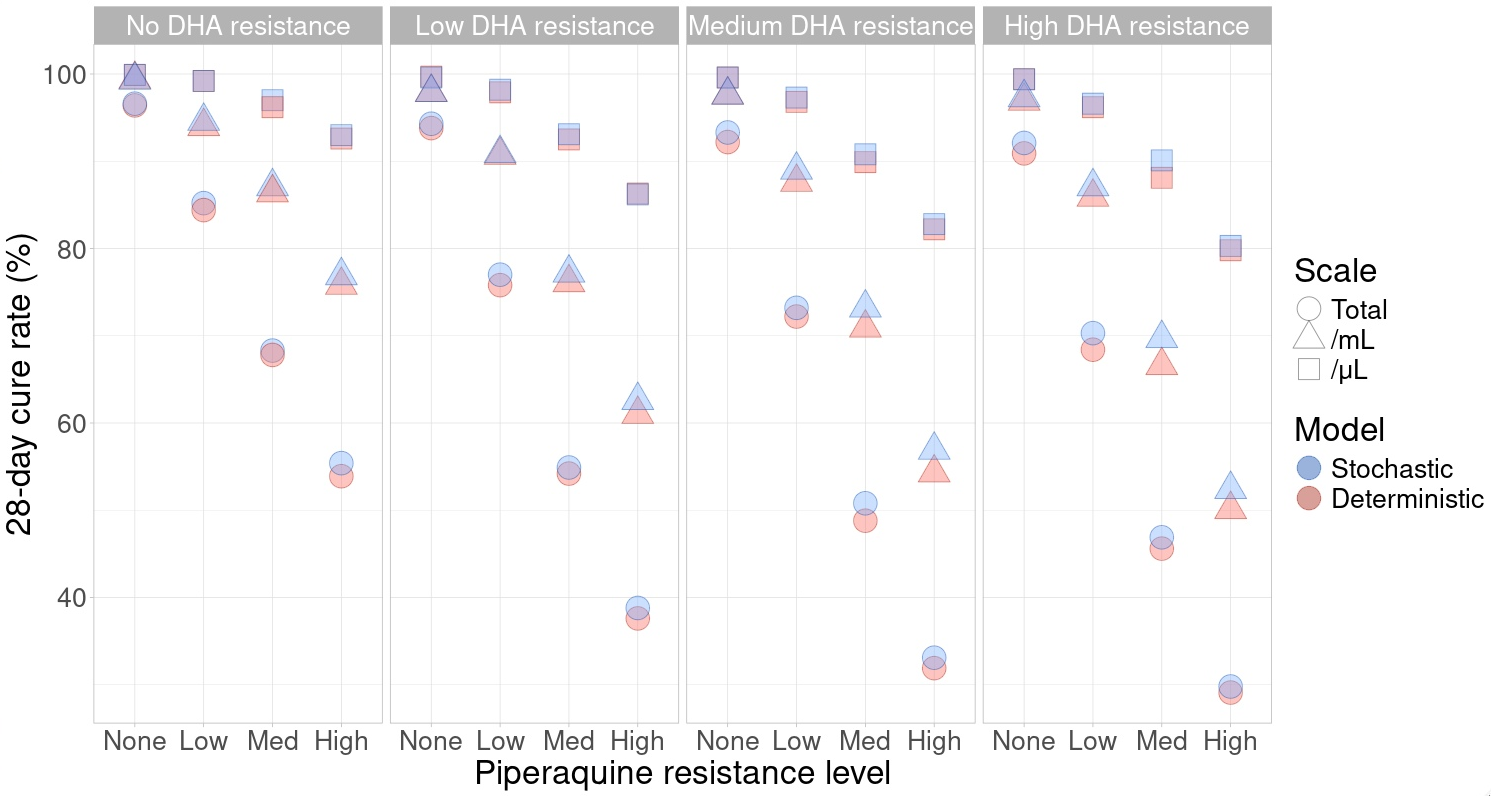


**References:**

1. Ezzet F, van Vugt M, Nosten F, Looareesuwan S, White NJ. Pharmacokinetics and pharmacodynamics of lumefantrine (benflumetol) in acute falciparum malaria. Antimicrob Agents Chemother 2000; 44: 697-704.

2. Jamsen KM, Duffull SB, Tarning J, Lindegardh N, White NJ, Simpson JA. Optimal designs for population pharmacokinetic studies of oral artesunate in patients with uncomplicated falciparum malaria. Malar J 2011; 10: 181.

3. Tarning J, Ashley EA, Lindegardh N, Stepniewska K, Phaiphun L, Day NPJ, McGready R, Ashton M, Nosten F, White NJ. Population Pharmacokinetics of Piperaquine after Two Different Treatment Regimens with Dihydroartemisinin-Piperaquine in Patients with Plasmodium falciparum Malaria in Thailand. Antimicrobial Agents and Chemotherapy 2008; 52: 1052-61.

4. Global Malaria Programme (GMP) GRC. WHO Guidelines for Malaria. In: World Health Organisation, 2024: 168.

5. White NJ, Pukrittayakamee S, Hien TT, Faiz MA, Mokuolu OA, Dondorp AM. Malaria. The Lancet 2014; 383: 723-35.

6. Dietz K, Raddatz G, Molineaux L. Mathematical model of the first wave of Plasmodium falciparum asexual parasitemia in non-immune and vaccinated individuals. Am J Trop Med Hyg 2006; 75: 46-55.

7. Zaloumis S, Humberstone A, Charman SA, Price RN, Moehrle J, Gamo-Benito J, McCaw J, Jamsen KM, Smith K, Simpson JA. Assessing the utility of an anti-malarial pharmacokinetic-pharmacodynamic model for aiding drug clinical development. Malar J 2012; 11: 303.

8. Dini S, Zaloumis S, Cao P, Price RN, Fowkes FJI, van der Pluijm RW, McCaw JM, Simpson JA. Investigating the Efficacy of Triple Artemisinin-Based Combination Therapies for Treating Plasmodium falciparum Malaria Patients Using Mathematical Modeling. Antimicrob Agents Chemother 2018; 62.

9. White NJ. Assessment of the pharmacodynamic properties of antimalarial drugs in vivo. Antimicrob Agents Chemother 1997; 41: 1413-22.

10. Pasay CJ, Rockett R, Sekuloski S, Griffin P, Marquart L, Peatey C, Wang CY, O'Rourke P, Elliott S, Baker M, Möhrle JJ, McCarthy JS. Piperaquine Monotherapy of Drug-Susceptible Plasmodium falciparum Infection Results in Rapid Clearance of Parasitemia but Is Followed by the Appearance of Gametocytemia. J Infect Dis 2016; 214: 105-13.

11. Simpson JA, Jamsen KM, Anderson TJ, Zaloumis S, Nair S, Woodrow C, White NJ, Nosten F, Price RN. Nonlinear mixed-effects modelling of in vitro drug susceptibility and molecular correlates of multidrug resistant Plasmodium falciparum. PLoS One 2013; 8: e69505.

12. Terkuile F, White NJ, Holloway P, Pasvol G, Krishna S. Plasmodium falciparum: In Vitro Studies of the Pharmacodynamic Properties of Drugs Used for the Treatment of Severe Malaria. Experimental Parasitology 1993; 76: 85-95.

13. Geary TG, Divo AA, Jensen JB. Stage specific actions of antimalarial drugs on Plasmodium falciparum in culture. Am J Trop Med Hyg 1989; 40: 240-4.

14. Taylor T, Olola C, Valim C, Agbenyega T, Kremsner P, Krishna S, Kwiatkowski D, Newton C, Missinou M, Pinder M, Wypij D. Standardized data collection for multi-center clinical studies of severe malaria in African children: establishing the SMAC network. Trans R Soc Trop Med Hyg 2006; 100: 615-22.

15. Howie SR. Blood sample volumes in child health research: review of safe limits. Bull World Health Organ 2011; 89: 46-53.
